# Analysis of Clinical and Laboratory Findings of Idiopathic Sudden Sensorineural Hearing Loss

**DOI:** 10.1038/s41598-020-63046-z

**Published:** 2020-04-08

**Authors:** Wen Xie, Qingqing Dai, Jianguo Liu, Yuehui Liu, Sten Hellström, Maoli Duan

**Affiliations:** 1 Department of Otolaryngology, Head and Neck Surgery, the Second Affiliated Hospital of Nanchang University, Nanchang, China; 2 Department of Otolaryngology, Head and Neck Surgery, Sichuan University West China Hospital, Chengdu, China; 3 Department of Otolaryngology, Head and Neck Surgery, Karolinska University Hospital, Karolinska Institute, Stockholm, Sweden; 4 Department of Clinical Science, Intervention and Technology, Karolinska Institute, Karolinska University Hospital, Stockholm, Sweden

**Keywords:** Neurological disorders, Biostatistics, Risk factors, Comorbidities

## Abstract

Idiopathic sudden sensorineural hearing loss (ISSNHL) is an emergency disease requiring immediate diagnosis and treatment. The incidence of ISSNHL in the Western countries’ population was estimated to 5–20 per 100,000 inhabitants. The etiology of ISSNHL remains unknown. Its pathogenesis is most often suggested to be due to a disturbed microcirculation and infection. Previous studies have reported that comorbidities, including hypertension, diabetes mellitus (DM), and hyperfibrinogenemia are risk factors of ISSNHL. This study aimed at investigating the clinical characteristics, laboratory parameters and comorbidities of patients with ISSNHL. Our study suggests that the annual incidence of ISSNHL in China mainland is 19 per 100 000. The clinical characteristics and prevalence of comorbidities of ISSNHL patients are different according to age distribution and hearing results. Moreover, the patients with vertigo, hypertension, DM and high TG suffered more often from severe hearing loss compared with the counterparts. This indicates that the cardiovascular and metabolic diseases (hypertension and hyperlipidemia) appeared to be closely associated with the occurrence and severity of ISSNHL.

## Introduction

Idiopathic sudden sensory neural hearing loss (ISSNHL) is characterized as an abrupt hearing loss of more than 30 dB in three contiguous frequencies within 72 hours^1^.

An earlier study reported that the incidence of ISSNHL in the Western countries’ population was estimated to 5–20 per 100,000 inhabitants^2^. More recent investigations showed the annual incidence of SSNHL to be 27 per 100,000 in the United States and 2.4 per 100 000 in western China, respectively^3,4^. Regarding age distribution, Rauch demonstrated that ISSNHL most frequently occurred in 43 to 53 years old patients^5^. On the other hand, a Japanese survey showed that ISSNHL was the most prevalent among patients aged 60–69 years old, and the hearing loss was the most severe in patients aged under 16 and over 65 years of age^6^.

ISSNHL is an emergency disease requiring immediate diagnosis and treatment. The pathogenesis of ISSNHL remains unknown, which influences the preventive and therapeutic strategy-making. Some pathophysiological mechanisms; including vascular disease, viral infection, metabolic disease, autoimmunity, trauma and combinations of multiple factors are suggested to be the causes of ISSNHL^7–9^. Recently, microcirculation disturbance has been hypothesized as the main etiology. The cochlea is supplied by the cochlear artery, a terminal artery without any collateral vessels to compensate for any occlusion of the blood vessel. Therefore, any disease interrupting the cochlear perfusion may eventually result in a reduction of the oxygen supply to cochlea and trigger ISSNHL. Cardiovascular and metabolic diseases such as hypertension, diabetes mellitus (DM) and hyperlipidemia, will reduce the elasticity of blood vessels and induce the formation of atherosclerosis, thus causing microangiopathy. Several studies have confirmed these findings, suggesting that these comorbidities are clinically associated with ISSNHL^10,11^.

Also, hyperfibrinogenemia has been assumed as a risk factor to ISSNHL, and the relationship between hyperfibrinogenemia and ISSNHL has been emphasized in several clinical and animal experiments^12,13^. The explanation of this association is that an elevation of fibrinogen can increase blood viscosity and generate vascular thrombosis, leading to an impaired regional blood supply, and consequently an increase of the possibility of onset of ISSNHL.

Against this background, we conducted the present retrospective study to estimate the incidence of ISSNHL in Chinese mainland inhabitants, by comparing the ISSNHL cases and the demographic data obtained from the Provincial Health committees. Besides, we aimed to examine the clinic characteristics of the patients, as well as audiometric features. We also examined the prevalence of comorbidities and abnormal laboratory test results and its relationship to age distribution and audiological results. Finally, we investigated the incidence of associated symptoms in ISSNHL patients and possible etiological factors that may cause ISSNHL.

## Materials and Method

### Patients

This retrospective study included patients with a diagnosis of ISSNHL consecutively hospitalized between August 2013 and December 2017 in the Second Affiliated Hospital of Nanchang University, a provincial tertiary referral hospital in southeast China.

The criterion for a diagnosis of ISSNHL has been elucidated in detail in previous statements^1^. Exclusion criteria included the following: those with hearing loss due to other causes such as otitis media, Meniere’s disease, otosclerosis, congenital deafness, presbycusis, vestibular schwannoma, and inner ear malformation. Patients with insufficient medical record data were also excluded.

**Figure 1:**
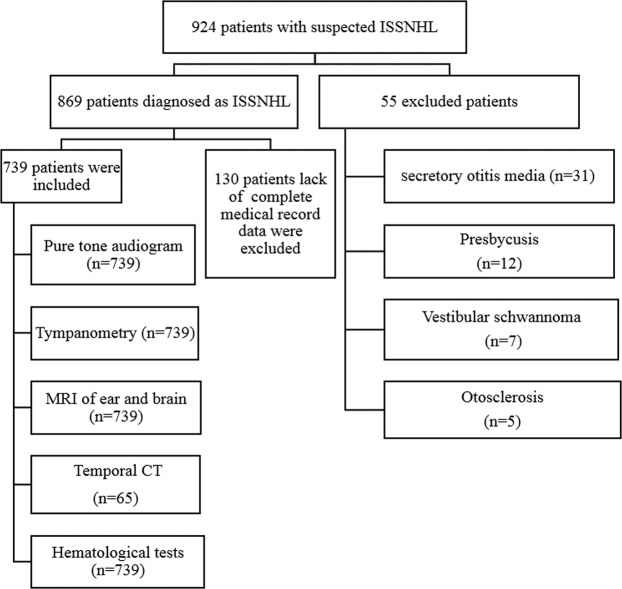
Flow gram of the study.

924 consecutive patients with suspected ISSNHL were examined by experienced audiologists when they were referred to our hospital. 869 of them were diagnosed as ISSNHL. Comparing this data with the demographic condition in the referral area, we estimated that the annual incidence of ISSNHL patients was roughly 19 per 100 000. Further 130 subjects were excluded due to lack of complete medical record data. Therefore, a total of 739 patients were included in this study (Fig. 1).

The study was done in accordance with the ethical principles and approved by the Second Affiliated Hospital of Nanchang University Institutional Review Board. Written, informed consent was obtained from all patients and/or their guardians.

### Test procedure

All patients underwent a detailed clinical interview. Clinical data, demographic information (collected from the provincial health commission), and past medical history were obtained. Routine general physical examination, general otorhinolaryngological examination, routine audiological and laboratory tests were undergone in all subjects. Temporal Computed Tomography (CT) scans were conducted in 65 patients, and Magnetic Resonance Imaging (MRI) scans of ear and brain were performed in all patients.

### Hearing evaluation

All patients’ hearing was evaluated with pure tone audiogram and tympanometry. All hearing tests were carried out by the same audiologist. Air and bone conduction were assessed at frequencies of 250 Hz, 500 Hz, 1 kHz, 2 kHz, 4 kHz, and 8 kHz.

Pure tone average (PTA) was calculated as the mean of air conduction thresholds at 0.5, 1, 2, and 4 kHz^10^. The hearing loss levels were categorized into 5 grades: mild (26–40 dB HL), moderate (41-55 dB HL), moderate to severe (56-70 dB HL), severe (71–90 dB HL), and profound (>90 dB HL)^14^. Audiogram patterns were classified into 5 types: ascending (the average threshold of 0.25–0.50 kHz was 20 dB higher than that of 4–8 kHz), descending (the average threshold of 4–8 kHz was 20 dB higher than that of 0.25–0.50 kHz), flat (all frequencies present similar thresholds and hearing threshold was below 80 dB HL), profound (all frequencies show similar threshold and hearing threshold was over 80 dB HL), and concave or convex type (average hearing degree of the mid-tone frequency was 20 dB higher than low and high frequencies)^15^.

### Hematological evaluation and comorbidities

Hematological tests were carried out in all patients. In addition to routine blood tests and biochemical tests, the parameters analyzed in this study were fasting blood sugar, hemostasis determinations, and lipid profile. The reference values defined as normal in our laboratory are listed in Table 1.

**Table 1:** Normal reference values of blood test parameters.

Blood test parameters	Normal reference values
Prothrombin time (PT)	9-13 s
Activated partial thromboplastin time (APTT)	20-40 s
Fibrinogen	2-4 g/L
Total cholesterol (TC)	<5.18 mmol/L
Triglycerides (TG)	<1.7 mmol/L
High density lipoprotein cholesterol (HDL-C)	Male: 1.16-1.42 mmol/L; Female: 1.29-1.55 mmol/L
Low-density lipoprotein cholesterol (LDL-C)	<3.1 mmol/L

Comorbidities including hypertension and diabetes mellitus (DM) were also assessed. Hypertension was defined as blood pressure ≥140/90 mmHg^16^ or previous physician-diagnosed hypertension. DM was diagnosed according to the consensus of the expert committee on the diagnosis and classification of DM^17^, or diagnosed by internists and were treated with antidiabetic medications.

### Statistical analysis

Categorical data were shown as percentages and compared using Chi-square test. Fisher’s exact test was used when expected counts in Chi-square test were insufficient. Ranked data were assessed through Mann-Whitney 2-sample test. All analyses were conducted using SPSS version 25 for Windows. All statistical tests were 2-sided, and statistically significant levels were set at 0.05 (P < 0.05).

## Result

### Clinical characteristics

Of the 739 patients with ISSNHL and complete medical records. 392 were males (53.0%) and 347 were females (47.0%). The mean age of all patients was 47 years (range: 4-82 years). ISSNHL affected right ear in 341 patients (46.1%), left ear in 378 patients (51.2%) and bilateral ears in 20 patients (2.7%).

### Age distribution

**Figure 2:**
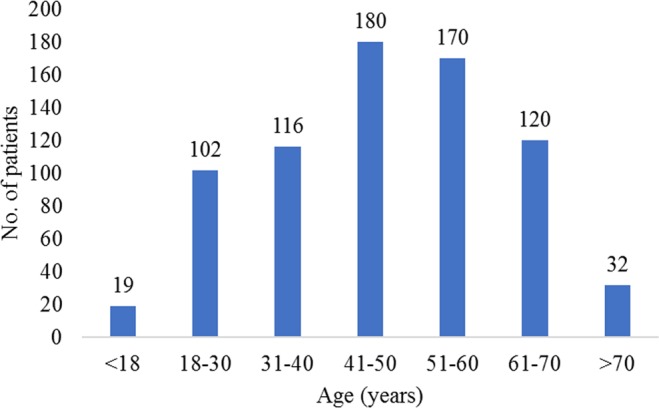
Age distribution in ISSNHL.

The peak age prevalence was in the group of patients 41–50 years of age, and the lowest prevalence was presented in the group under-18 years of age (Fig. 2). The clinical characteristics based on various age groups are listed in Table 2. Younger patients were more predisposed to mild and ascending hearing loss, whereas, older patients were more susceptible to the flat-hearing-type and more severe hearing loss.

**Table 2:** Characteristics of ISSNHL based on age groups (n = 759 ears, n (%)).

Characteristics	Under-18	18-30	31-40	41-50	51-60	61-70	over-70	statistical values*	P
	age group	age group	age group	age group	age group	age group	age group		
	(n = 19)	(n = 104)	(n = 120)	(n = 186)	(n = 175)	(n = 121)	(n = 34)		
Male	12 (63.2)	58 (55.8)	61 (50.8)	105 (56.5)	101 (57.7)	53 (43.8)	13 (38.2)	10.869	0.092
Female	7 (36.8)	46 (44.2)	59 (49.2)	81 (43.5)	74 (42.3)	68 (56.2)	21 (61.8)		
Unilateral hearing loss	19 (100)	100 (98.0)	112 (96.6)	174 (96.7)	165 (97.1)	119 (99.2)	30 (93.8)	4.214	0.583
Bilateral hearing loss	0 (0)	2 (2.0)	4 (3.4)	6 (3.3)	5 (2.9)	1 (0.8)	2 (6.3)		
**Accompanying symptoms**									
Tinnitus	18 (94.7)	103 (99)	119 (99.2)	178 (95.7)	170 (97.1)	115 (95.0)	33 (97.1)	6.865	0.270
Vertigo	1 (5.3)	9 (8.7)	8 (6.7)	26 (14.0)	27 (15.4)	17 (14.0)	2 (5.9)	9.559	0.144
BPPV	0 (0)	2 (1.9)	3 (2.5)	4 (2.2)	5 (2.9)	6 (5.0)	0 (0)	2.853	0.797
**Audiogram curve types**									
Ascending	3 (15.8)	18 (17.3)	27 (22.5)	19 (10.2)	7 (4.0)	4 (3.3)	1 (2.9)	41.007	0.000
Descending	1 (5.3)	11 (10.6)	16 (13.3)	12 (6.5)	16 (9.1)	10 (8.3)	0 (0)	8.487	0.205
Flat	3 (15.8)	31 (29.8)	30 (25.0)	69 (37.1)	58 (33.1)	52 (43.0)	17 (50.0)	16.945	0.009
Profound	10 (52.6)	43 (41.3)	46 (38.3)	82 (44.1)	92 (52.6)	54 (44.6)	16 (47.1)	7.327	0.292
Concave or convex	2 (10.5)	1 (1.0)	1 (0.8)	4 (2.2)	2 (1.1)	1 (0.8)	0 (0)	7.377	0.190
**Hearing level**									
Mild	5(26.3)	19 (18.3)	29 (24.2)	27 (14.5)	12 (6.9)	8 (6.6)	1 (2.9)	31.690	0.000
Moderate	1 (5.3)	14 (13.5)	20 (16.7)	25 (13.4)	26 (14.9)	13 (10.7)	4 (11.8)	3.268	0.775
Moderate to severe	1 (5.3)	14 (13.5)	10 (8.3)	32 (17.2)	27 (15.4)	31 (25.6)	8 (23.5)	17.183	0.009
Severe	3 (15.8)	24 (23.1)	29 (24.2)	42 (22.6)	40 (22.9)	32 (26.4)	9 (26.5)	1.540	0.957
Profound	9 (47.4)	33 (31.7)	32 (26.7)	60 (32.3)	70 (40.0)	37 (30.6)	12 (35.3)	7.327	0.292
**comorbidities**									
Hypertension	2 (10.5)	6 (5.8)	11(9.2)	36(19.4)	56 (32)	46 (38.0)	17 (50.0)	71.033	0.000
Diabetes	1 (5.3)	0 (0)	1 (0.8)	12 (6.5)	16 (9.1)	10 (8.3)	2 (5.9)	17.561	0.007
**Abnormal blood coagulation**									
High fibrinogen	0 (0)	4 (3.8)	1 (0.8)	4 (2.2)	6 (3.4)	8 (6.6)	2 (5.9)	7.991	0.185
Reduced APTT	0 (0)	7 (6.7)	4 (3.3)	6 (3.2)	17 (9.7)	8 (6.6)	1 (2.9)	10.697	0.098
Reduced PT	0 (0)	1 (1.0)	3 (2.5)	5 (2.7)	4 (2.3)	2 (1.7)	0 (0)	1.435	0.963
**High blood lipid**									
High TC	0 (0)	24 (23.1)	37 (30.8)	74 (39.8)	85 (48.6)	51 (42.1)	13 (38.2)	33.624	0.000
High TG	0 (0)	5 (4.8)	24 (20.0)	42 (22.6)	32 (18.3)	32 (26.4)	9 (26.5)	25.374	0.000
Low HDL	5 (26.3)	35 (33.7)	51 (42.5)	81 (43.5)	62 (35.4)	56 (46.3)	18 (52.9)	10.247	0.115
High LDL	2 (10.5)	32 (30.8)	46 (38.3)	87 (46.8)	95 (54.3)	52 (43.0)	11 (32.4)	27.354	0.000

*The Chi-squared test or Fisher’s exact test.

The evaluation of comorbidities and laboratory tests revealed a higher percentage of hypertension, DM, high total cholesterol (TC), high triglycerides (TG) and high low-density lipoproteins (LDL) in the older age group patients than in younger age group patients.

### Audiological characteristic

**Figure 3:**
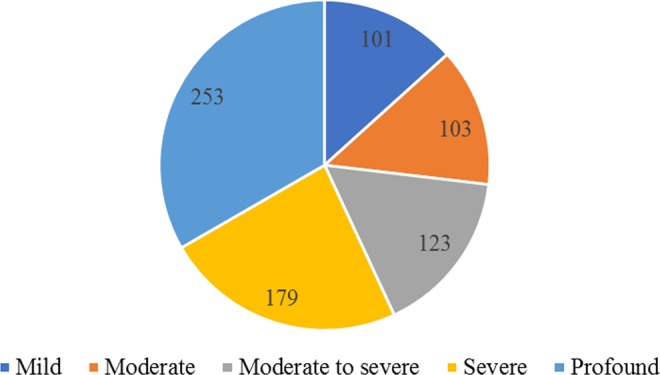
The numbers of affected ears in different hearing level group.

Among 739 patients with ISSNHL, 719 patients had unilateral and 20 had bilateral ISSNHL. 101 (13.3%) ears were classified as mild, 103 (13.6%) as moderate, 123 (16.2%) as moderate to severe, 179 (23.6%) as severe, and 253 (33.3%) as profound ISSNHL (Fig. 3). Patients’ characteristics related to hearing loss severity are shown in Table 3. Patients with vertigo, hypertension and high TG suffered from more severe hearing loss (the mean ranks of these groups were higher) than the counterparts.

**Table 3:** Characteristics of ISSNHL based on hearing levels (n = 759 ears, n (%)).

Characteristics	Mild	Moderate	Moderate to severe	Severe	Profound	Z	P
	(n = 101)	(n = 103)	(n = 123)	(n = 179)	(n = 253)		
Male	43 (42.6)	56 (54.4)	66 (53.7)	110 (61.5)	128 (50.6)	−0.708	0.479
Female	58 (57.4)	47 (45.6)	57 (46.3)	69 (38.5)	125 (49.4)		
Unilateral hearing loss	96 (95.0)	91 (88.3)	115 (93.5)	173 (96.6)	244 (96.4)	−2.125	0.034
Bilateral hearing loss	5 (5.0)	12 (11.7)	8 (6.5)	6 (3.4)	9 (3.6)		
**Accompanying symptoms**							
Tinnitus	98 (97.0)	101 (98.1)	120 (97.6)	175 (97.8)	242 (95.7)	−1.143	0.253
Vertigo	7 (6.9)	7 (6.8)	8 (6.5)	20 (11.2)	48 (19.0)	−4.266	0.000
BPPV	1 (1.0)	1 (1.0)	3 (2.4)	5 (2.8)	10 (4.0)	−1.936	0.053
**comorbidities**							
Hypertension	16 (15.8)	28 (27.2)	20 (16.3)	41 (22.9)	69 (27.3)	−2.06	0.039
Diabetes	0 (0)	4 (3.9)	5 (4.1)	16 (8.9)	17 (6.7)	−2.55	0.011
**Abnormal blood coagulation**							
High fibrinogen	1 (1.0)	4 (3.9)	3 (2.4)	6 (3.4)	11 (4.3)	−1.364	0.173
Reduced APTT	3 (3.0)	5 (4.9)	5 (4.1)	11 (6.1)	19 (7.5)	−1.862	0.063
Reduced PT	2 (2.0)	6 (5.8)	1 (0.8)	1 (0.6)	5 (2.0)	−1.02	0.308
**High blood lipid**							
High TC	37 (36.6)	39 (37.9)	45 (36.6)	71 (39.7)	92 (36.4)	−0.089	0.929
High TG	14 (13.9)	16 (15.5)	18 (14.6)	36 (20.1)	60 (23.7)	−2.709	0.007
Low HDL	31 (30.7)	45 (43.7)	50 (40.7)	69 (38.5)	113 (44.7)	−1.748	0.081
High LDL	44 (43.6)	47 (45.6)	53 (43.1)	81 (45.3)	100 (39.5)	−0.997	0.319

Data were analyzed by non-parametric Mann-Whitney U test.

**Figure 4:**
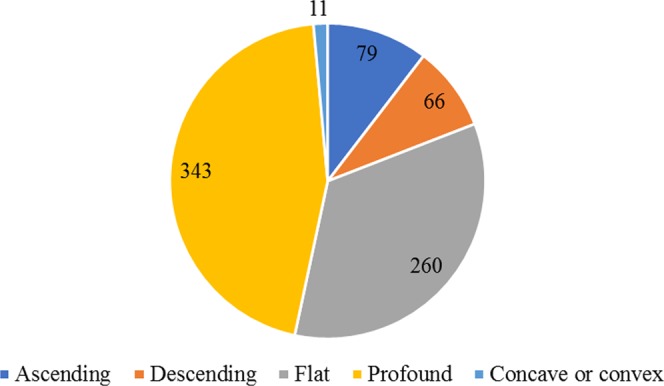
The numbers of affected ears in different audiogram type group.

In terms of the audiogram shape, as depicted in Fig. 4, the most common one was profound (45.2%), followed by flat (34.3%), ascending (10.4%) and descending type (8.7%). Concave and convex hearing loss was rare (1.4%).

The percentages of patients with vertigo in patients with profound hearing loss were significantly higher than those with other types of hearing loss. The correlation between hearing configuration and the clinic characteristics of the patients is shown in Table 4.

**Table 4:** Characteristics of ISSNHL based on audiogram types (n = 759 ears, n (%)).

Characteristics	Ascending	Descending	Flat	Profound	Concave or convex	statistical values*	P
	(n = 79)	(n = 66)	(n = 260)	(n = 343)	(n = 11)		
Male	35 (44.3)	43 (65.2)	137 (52.7)	182 (53.1)	6 (54.5)	6.330	0.176
Female	44 (55.7)	23 (34.8)	123 (47.3)	161 (46.9)	5 (45.5)		
Unilateral hearing loss	76 (96.2)	58 (87.9)	241 (92.7)	333 (97.1)	11 (100)	11.683	0.014
Bilateral hearing loss	3 (3.8)	8 (12.1)	19 (7.3)	10 (2.9)	0 (0)		
**Accompanying symptoms**							
Tinnitus	78 (98.7)	65 (98.5)	255 (98.1)	328 (95.6)	10 (90.9)	5.639	0.179
Vertigo	3 (3.8)	7 (10.6)	20 (7.7)	60 (17.5)	0 (0)	21.226	0.000
BPPV	0 (0)	3 (4.5)	4 (1.5)	13 (3.8)	0 (0)	5.919	0.155
**comorbidities**							
Hypertension	16 (20.3)	10 (15.2)	57 (21.9)	90 (26.2)	1 (9.1)	6.047	0.196
Diabetes	0 (0)	3 (4.5)	15 (5.8)	24 (7.0)	0 (0)	7.308	0.101
**Abnormal blood coagulation**							
High fibrinogen	0 (0)	2 (3.0)	9 (3.5)	14 (4.1)	0 (0)	3.419	0.401
Reduced APTT	3 (3.8)	2 (3.0)	12 (4.6)	26 (7.6)	0 (0)	3.543	0.431
Reduced PT	1 (1.3)	4 (6.1)	4 (1.5)	6 (1.7)	0 (0)	4.980	0.231
**High blood lipid**							
High TC	27 (34.2)	22 (33.3)	99 (38.1)	134 (39.1)	2 (18.2)	3.009	0.556
High TG	13 (16.5)	8 (12.1)	43 (16.5)	79 (23.0)	1 (9.1)	7.719	0.102
Low HDL	25 (31.6)	28 (42.4)	110 (42.3)	141 (41.1)	4 (36.4)	3.151	0.533
High LDL	31 (39.2)	34 (51.5)	109 (41.9)	148 (43.1)	3 (27.3)	3.638	0.457

*The Chi-squared test or Fisher’s exact test.

### Hematological examination

There were 276 patients (37.3%) with an increased level of TC; 143 cases (19.4%) with increased TG; 300 patients (40.6%) with decreased high-density lipoprotein (HDL); and 316 patients (42.8%) with elevated LDL. High plasma fibrinogen was found in 24 patients (3.2%), shortened activated partial thromboplastin time (APTT) and prothrombin time (PT) were present in 42 (5.7%) and 13 (1.8%) patients, respectively.

### MRI results

All 739 patients’ MRI of ear had normal results. Regarding brain MRI, 493 patients’ results were normal, but old **s**light ischemia was visualized in 246 patients, and 42 of them had mild brain atrophy. All these 42 patients were over 50 years old.

### Comorbidities

Among the 739 patients, 168 patients (22.7%) suffered from hypertension, and 41 (5.5%) patients had a comorbidity of DM.

### Associated symptoms and possible ethological factors

Some of the patients presented with associated symptoms such as tinnitus (97%), spontaneous vertigo (duration 1–48 h, 11.8%), and BPPV (2.6%). The percentage of patients complaining of vertigo was higher in patients with profound hearing loss than those with milder hearing loss. Among the 739 patients, 647 patients (87.6%) were identified with no obvious causative factors, 30 patients (4.1%) with upper respiratory infections, 31 patients (4.2%) with fatigue, 22 patients (3.0%) with psychosocial stress, 3 patients with excessive noise-exposure, and a 9-year-old boy suffered from mumps before the onset of ISSNHL.

## Discussion

Our study corroborates previous findings that the annual incidence of patients with ISSNHL, is 5-20 per 100,000^2^. Their mean age is ranging from 45 to 55 years, there is an overall slight male preponderance, and the highest prevalence of ISSNHL occurs in the age group of 41–50 years^6,18,19^. In our study, the prevalence of idiopathic bilateral ISSNHL was 2.7%, which is lower than that of 8.6% reported earlier^19^. It is generally accepted that a low frequency hearing loss of ISSNHL may indicate a sign of hydrops^18^, as seen in Meniere disease. Thus a low frequency hearing impairment may represent a partial symptom of Meniere’s disease. However, no case in our study presented with fluctuating hearing loss and recurrent vertigo, so they were diagnosed as ISSNHL instead of Meniere’s disease.

Our results showed that the prevalence of mild ascending hearing loss was higher in younger patients, and flat hearing loss was more common among older patients. Regarding these findings one could speculate that the theory of blood circulation disturbance might be the etiology of some cases of ISSHL. A transient reduction in blood pressure values, commonly occurs in young subjects without vascular risk factors, which may cause cochlear ischemia and reversible hearing impairment, and restoration^20,21^. Previous studies reported a strong relationship between systemic hypotension and sensorineural hearing loss affecting lower frequencies among young patients^21,22^. One proposed mechanism for this phenomenon is that the terminal cochlear artery which supplies the blood to the apical turn is most vulnerable to a transient insufficiency of blood flow^22^.

The prevalence of hypertension among ISSNHL patients was 22.7%, which was a little higher than that of the local population (19.8%)^23^. Moreover, the DM prevalence in ISSNHL patients in our study was 5.5%, which is lower than recently reported data in literature (9.7%)^24^. Regarding age distribution and comorbidities and laboratory test parameters, it is understandable that in our study, among ISSNHL patients, the prevalence of cardiovascular and metabolic diseases increased with their age growing.

We analyzed the correlation between audiogram profile and clinical characteristics and found that the distribution of audiogram types based on hypertension, DM, hyperlipidemia and hyperfibrinogenemia did not show any statistical differences. However, patients with hypertension, DM and high TG group had a more pronounced hearing loss compared to their counterparts. Our study also for the first-time reports that patients with hypertension are more likely to suffer from a more pronounced hearing loss. It is however worth mentioning that one earlier report found a positive correlation between higher levels of systolic blood pressure and age-related hearing loss in the speech frequencies^25^. Another study demonstrated the hearing loss level to be higher in patients with than without metabolic syndrome, but the difference was not statistically significant^26^. Similar results were observed in terms of audiogram patterns. However, that study did not evaluate the relationship of each specific metabolic disease with the severity of hearing loss. Liang, *et al*. revealed a significantly higher probability of severe hearing loss in the patients with vertigo/dizziness, diabetes mellitus, heart disease, or those aged 65 years and older^6^. In addition, another study reported that in comparison with healthy controls, higher levels of blood glucose, glycated hemoglobin (HbA1C), lipoprotein (a), and factor VIII were found among ISSNHL patients. The moderate-severe ISSNHL (PTA > 40 dB HL) patients showed higher significant values of blood glucose, glycated hemoglobin (HbA1C), uric acid, factor VIII, and homocysteine than mild ISSNHL (PTA ≤ 40 dB HL) patients^10^. All these laboratory parameters reflect the patients’ metabolic and thrombophilic states and are considered to be the possible factors involved in a vascular etiology of ISSNHL. Our study thus supports the concept of vascular hypothesis for ISSNHL and its severity. The comorbidities, including hypertension, DM and dyslipidemia, may induce atherosclerotic changes, and result in cochlear microcirculation disturbance. Specifically, animal experiments have demonstrated that dyslipidemia can cause ISSNHL by affecting the inner ear blood supply^27,28^.

In agreement with previous studies^4,29^, we found that the patients with vertigo were more susceptible to a more pronounced hearing loss. There are no previous reports which explain this phenomenon yet. Current evidence however shows that ISSNHL patients with vertigo had a higher risk of vestibular organ lesions^30^ and that this may be negatively associated with hearing recovery^31^. Therefore, ISSNHL patients with vertigo have a worse prognosis and we emphasize the importance of immediate and comprehensive treatments, such as medical treatments and vestibular rehabilitation.

The major limitation of this study is that we do not compare the clinic data of ISSNHL patients to a control group. Further studies will be needed to clarify the difference of clinic and laboratory results between ISSNHL patients and normal people.

## Conclusion

Our study demonstrates that the age distribution and clinical characteristics of ISSNHL patients vary according to levels of hearing loss and audiogram types. Moreover, ISSNHL patients with vertigo tend to suffer from a more severe hearing loss.

## Data Availability

All data generated or analyzed during this study are included in this published article.
